# Epstein-Barr Virus miR-BART17-5p Promotes Migration and Anchorage-Independent Growth by Targeting Kruppel-Like Factor 2 in Gastric Cancer

**DOI:** 10.3390/microorganisms8020258

**Published:** 2020-02-15

**Authors:** Jae Hee Yoon, Kyoungmi Min, Suk Kyeong Lee

**Affiliations:** Department of Medical Life Sciences, Department of Biomedicine & Health Sciences, College of Medicine, The Catholic University of Korea, Seoul 06591, Korea; yjh823@catholic.ac.kr (J.H.Y.); kyoungmimin@gmail.com (K.M.)

**Keywords:** Epstein-Barr virus, stomach neoplasms, microRNAs, KLF2, cell migration, tumor suppressor

## Abstract

Epstein-Barr virus (EBV) infects more than 90% of the global population and is associated with a variety of tumors including nasopharyngeal carcinoma, Hodgkin lymphoma, natural killer/T lymphoma, and gastric carcinoma. In EBV-associated gastric cancer (EBVaGC), highly expressed EBV BamHI A rightward transcripts (BART) miRNAs may contribute to tumorigenesis with limited viral antigens. Despite previous studies on the targets of BART miRNAs, the functions of all 44 BART miRNAs have not been fully clarified. Here, we used RNA sequencing data from the Cancer Genome Atlas to find genes with decreased expression in EBVaGC. Furthermore, we used AGS cells infected with EBV to determine whether expression was reduced by BART miRNA. We showed that the expression of Kruppel-like factor 2 (*KLF2*) is lower in AGS-EBV cells than in the AGS control. Using bioinformatics analysis, four BART miRNAs were selected to check whether they suppress *KLF2* expression. We found that only miR-BART17-5p directly down-regulated *KLF2* and promoted gastric carcinoma cell migration and anchorage-independent growth. Our data suggest that *KLF2* functions as a tumor suppressor in EBVaGC and that miR-BART17-5p may be a valuable target for effective EBVaGC treatment.

## 1. Introduction

Gastric cancer (GC) is the fifth most common cancer worldwide with a high incidence in East Asia. In South Korea, GC has a higher incidence rate than in other countries [1]. Despite efforts to treat advanced GC, therapies remain an active area of investigation because the causes and pathology of GC are diverse [2]. Therefore, GC needs to be studied at the molecular level.

The Cancer Genome Atlas (TCGA) has classified GC into four subtypes: Epstein-Barr virus (EBV), microsatellite instability (MSI), chromosomal instability (CIN), and genomically stable (GS). EBV GC accounts for 9% of all GC [3]. EBV infects epithelial cells through various mechanisms [4,5]. EBV GC is characterized by the hypermethylation of CpG islands in the promoter and the downregulation of tumor suppressor genes [6]. EBV has a modified latency 1 infection expressing EBV-encoded small RNAs (EBERs), Epstein-Barr nuclear antigen 1 (*EBNA1*), latent membrane protein 2A (*LMP2A*), and BamHI A rightward transcripts (BART) microRNAs (miRNAs) in EBV-associated GC (EBVaGC) [7]. As a limited number of viral proteins are expressed, it would be of interest to identify the role of BART miRNAs expressed at a high level in EBVaGC.

MicroRNA consists of 19–23 nucleotides single-stranded noncoding RNA that binds to the 3′UTR of mRNA and inhibits the translation of the target protein. EBV BART miRNAs have been reported to regulate the expression of viral genes *BZLF1*, *BRLF1*, and *LMP1*, thereby modulating the life cycle of EBV [8,9]. Microarray analysis showed that the expression of cellular genes was dramatically different between EBV infected and uninfected GC cells [10]. Gottwein et al. [11] reported that EBV BART miRNAs are likely to target multiple cellular genes. For this reason, BART miRNAs regulate cellular gene expression [12,13,14,15]. Furthermore, tumorigenesis is promoted when BART miRNAs target tumor suppressor genes [16]. Despite some studies on the targets of BART miRNAs, the functions of all 44 BART miRNAs have not been clarified and further studies are required.

Among the BART miRNAs, miR-BART17-5p is known to be highly expressed and associated with poor prognosis in nasopharyngeal carcinoma (NPC) [17,18]. In diffuse large B-cell lympoma, miR-BART17-5p is associated with poor prognosis, reduces sensitivity to cisplatin [9], and targets BCL6 to promote *NF-κB* [19]. miR-BART17-5p is also expressed in EBVaGC at substantial levels [20,21], but the function of miR-BART17-5p in EBVaGC is still unclear.

The Kruppel-like factor (KLF) family consists of 17 members and belongs to DNA-binding transcriptional regulators. KLFs function as suppressors or oncogenes in tumorigenesis [22]. KLFs are also associated with EBV. The expression of *KLF5* is increased by the *ED-L2* promoter of the EBV, leading to increased proliferation in esophageal epithelia [23]. In epithelial cells, *KLF4* binds to the promoters of EBV immediate-early genes (*BZLF1* and *BRLF1*) to increase lytic replication [24]. However, there are insufficient studies on the function of the KLF family as a tumor suppressor in EBVaGC. Therefore, we analyzed RNA sequencing data from TCGA to select KLFs that act as tumor suppressors in EBVaGC. To investigate tumor suppressors, we focused on five KLFs which are suppressed in GC tissues compared to normal tissues. In fact, previous reports show that most of them act as tumor suppressors in GC [25,26]. We found that the expression of *KLF2* was most reduced in EBVaGC.

*KLF2*, a member of the KLF family, acts as a tumor suppressor in many cancers [27]. *KLF2* is expressed at a low level in non-small cell lung cancer and pancreatic cancer [28,29]. Likewise, *KLF2* is suppressed in GC [30,31,32]. The expression of *KLF2* was reduced by miR-32-5p in GC [33]. Furthermore, *KLF2* expression in GC was down-regulated by several lncRNAs, resulting in increased tumorigenesis [34,35,36,37]. However, there are few reports that investigate the association between EBVaGC and *KLF2*. As *KLF2* is known to be regulated by noncoding RNAs, we investigated whether *KLF2* is regulated by EBV BART miRNAs. We also have investigated whether *KLF2* acts as a tumor suppressor in EBVaGC and affects tumorigenesis.

In this study, we demonstrated that miR-BART17-5p directly down-regulated *KLF2*, promoting cell migration and anchorage-independent growth. Our data suggest that BART miRNAs may contribute to the tumorigenesis of EBVaGC.

## 2. Materials and Methods

### 2.1. Cell Lines and Culture Conditions

AGS, SNU-719, MKN-1, MKN-28, NCI-N87, SNU484, and NCC24 cells were purchased from Korean Cell Line Bank (Seoul, Korea). YCCEL1 cells were given by professor Sun Young Rha (Yonsei University College of Medicine, Republic of Korea). AGS-EBV cells were given by Takada K (Institute for Genetic Medicine, Hokkaido University, Japan). The GC cell lines were cultured in RPMI 1640 (Gibco, Grand Island, NY, USA) containing 10% fetal bovine serum (FBS, Corning, NY, USA), 100 U/mL penicillin, and 100 μg/mL streptomycin (Gibco) except for YCCEL1 which was cultured in Eagle’s minimal essential medium (Lonza Benelux BV, Breda, the Netherlands). AGS-EBV is an AGS cell line infected with a recombinant Akata strain of EBV [38]. To culture AGS-EBV cells, 400 μg/mL of G418 (Gibco) was added to the medium. SNU-719 [39], YCCEL1 [40], and NCC-24 [41] are gastric carcinoma cell lines naturally infected with EBV. The human embryonic kidney cell line HEK293T was cultured in Dulbecco’s modified Eagle’s medium (DMEM, Gibco), supplemented with 10% FBS, 100 U/mL penicillin, and 100 μg/mL streptomycin (Gibco). All cells were incubated at 37 °C and supplemented with 5% CO_2_.

### 2.2. Target Prediction 

The *KLF2* sequence was obtained from the National Center for Biotechnology Information (NM_016270.3). To examine whether the 3′UTR of *KLF2* can be targeted by BART miRNAs, we used the publicly available RNA hybrid program (http://bibiserv.techfak.uni-bielefeld.de/rnahybrid/).

### 2.3. Transfection of BART miRNA Mimics and Inhibitors

All BART miRNA mimics were purchased from Genolution Pharmaceuticals (Seoul, South Korea). A control inhibitor and a miR-BART17-5p inhibitor were purchased from Invitrogen (Carlsbad, CA, USA). All transfection experiments were performed using Lipofectamine 2000 (Invitrogen) according to the manufacturer’s protocol. Protein and RNA were extracted 48 h after transfection.

### 2.4. Quantitative Reverse Transcription-PCR (qRT-PCR)

Cells were harvested and total RNA was extracted using RNAisoplus (TaKaRa, Tokyo, Japan) reagent according to the manufacturer’s instruction. cDNA was synthesized using 1.5 µg total RNA, oligo(dT) primers (Macrogen, Seoul, South Korea) and Moloney murine leukemia virus (M-MLV) reverse transcriptase (Invitrogen). Real-time PCR was carried out using a SYBR Green qPCR kit (TaKaRa) with an Mx3000p real-time PCR system (Stratagene, La Jolla, CA, USA). The sequences of the primers used for each gene were as follows: *KLF2*, 5′-GCAAGACCTACACCAAGAGTTCG-3′ and 5′-CATGTGCCGTTTCATGTGC-3′; *GAPDH*, 5′-ATGGGGAAGGTGAAGGTCG-3′ and 5′-GGGGTCATTGATGGCAACAATA-3′. PCR conditions were 95 °C for 5 min, followed by 40 cycles at 95 °C for 30 s, 60 °C for 30 s, and 72 °C for 30 s. Relative gene expression was calculated by the comparative Ct method using *GAPDH* as an internal loading control.

### 2.5. Western Blot Analysis

Cell lysate was subjected to 12% sodium dodecyl sulfate (SDS) polyacrylamide gel electrophoresis, and the separated proteins were transferred to a polyvinylidene fluoride (PVDF) membrane. Rabbit polyclonal antibody against *KLF2* (ab139699, Abcam, Cambridge, MA, USA) and *p21* (C-19, Santa Cruz Biotechnology, Santa Cruz, CA, USA) were used as primary antibodies. Secondary antibody was purchased from Cell Signaling (Beverly, MA, USA). Protein bands were visualized using an enhanced chemiluminescence detection system.

### 2.6. Plasmid Constructs

The full-length 3′UTR of *KLF2* mRNA was amplified from the gDNA of AGS cells. The amplified PCR product containing XhoI and NotI sites at each end was inserted between the Renilla luciferase coding sequence and the poly(A) site of the psiCHECK-2 plasmid (Promega, Madison, WI, USA) to produce psiC-KLF2. The primers for the 3′UTR of *KLF2* containing XhoI and NotI sites are as follows: 5′-TCTAGGCGATCGCTCGAGCCGGGACGCCCCCGCCCA-3′ and 5′-TTATTGCGGCCAGCGGCCGCCTCGGAAAATGAATCAGATTGTCA-3′. Mutations were introduced into the two seed match sequences of psiC_KLF2 to produce psiC_KLF2_M1, psiC_KLF2_M2, and psiC_KLF2_M1M2 using an EZ change site-directed mutagenesis kit (Enzynomics, Daejeon, South Korea).

### 2.7. Luciferase Reporter Assay

To investigate the direct effects of BART miRNAs on the expression of *KLF2*, HEK293T and AGS cells were seeded in a 96-well plate at 5.5 × 10^3^ cells/well. After 24 h, cells were co-transfected with psiC-KLF2 and the BART miRNA mimic using Lipofectamine 2000 (Invitrogen). Luciferase activity was measured 48 h post-transfection using the Dual-Glo luciferase reporter assay system (Promega). For each sample, Renilla luciferase activity was normalized using the internal control firefly luciferase activity. 

### 2.8. Wound Healing Assay

To evaluate the effect of miR-BART17-5p on cell migration, AGS (1 × 10^6^ cells/well) and AGS-EBV (1.5 × 10^6^) cells were each seeded into six-well plates and allowed to reach 90–95% confluence. A monolayer of cells covering the plate was scratched with a sterile 200 µL pipette tip and subsequently washed with phosphate-buffered saline to remove cell debris. The cells were transfected with miR-BART17-5p, miR-BART17-5p inhibitor, or siKLF2. The cells were then cultured in RPMI-1640 medium containing 0.1% FBS at 37 °C in an incubator with 5% CO_2_. The scratched wounds were observed by an Axiovert 200 (Carl Zeiss, Thornwood, NY, USA) microscope just after transfection (time 0) and 48 h after transfection. Photographs were taken to assess the level of migration in each group of transfected cells, and wound areas were measured by Image J 1.37v software (National Institutes of Health, Bethesda, MD, USA). Three independent experiments were performed.

### 2.9. Soft Agar Colony Formation Assay

To confirm if *KLF2* was involved in anchorage-independent growth, a soft agar colony formation assay was performed. AGS and AGS-EBV were each transfected with 30 nM miR-BART17-5p mimic or inhibitor. After 24 h, the cells were harvested and seeded (3000 cells/well) in 0.6% Bacto Agar (214010; BD, Franklin Lakes, NJ, USA) mixed with culture medium (on top of 1% agar with medium) in six-well plates. Cells were cultured at 37 °C for 4–6 weeks. The images of the plates were captured under a microscope and camera. Then, the pictures were analyzed using Image J software. Three independent experiments were performed.

### 2.10. MTT Assay (Cell Viability Assay)

Cell proliferation was analyzed by using a 3-(4,5-dimethylthiazol-2-yl)-2,5-diphenyltetrazolium bromide (MTT) assay (Amresco, Shanghai, China). AGS cells (3 x10^3^) were seeded in 96-well plates. At a set time following transfection, 20 µl of MTT solution (5 mg/mL) was added to each well. After 4 h, the medium and MTT solution were removed. Then, 100 µl DMSO (Sigma-Aldrich, St. Louis, MO, USA) was added to each well. The absorbance at 595 nm was measured with SoftMax apparatus (Molecular Devices, Sunnyvale, CA, USA) 24 h after adding the DMSO.

### 2.11. KLF2 Knockdown by Small Interfering RNA (siRNA)

AGS cells were transfected with 30 nM KLF2-specific siRNA (siKLF2; Bioneer, Daejon, South Korea) to knockdown *KLF2*. The sequence of the siKLF2 was 5′-GAGACAGGUGGGCAUUUUU-3′ and the sequence of the control siRNA was 5′-CCUCGUGCCGUUCCAUCAGGUAGUU-3′. Protein and RNA were extracted 48 h after transfection.

### 2.12. KLF2 Overexpression Vector

A human *KLF2* expression vector (#60441, Addgene, Cambridge, MA, USA) was used for migration and soft agar colony formation assays.

### 2.13. Propidium Iodide (PI) Staining

AGS cells were trypsinized, washed twice with cold PBS and fixed in 70% ethanol at −20 °C overnight. The fixed cells were resuspended in PBS containing 20 µg/mL RNase A (Invitrogen) and 2.5 µg/mL PI (Sigma-Aldrich). The distribution of cells in each phase of the cell cycle was analyzed using FACSCalibur apparatus (BD Biosciences, San Diego, CA, USA) as described previously [42].

### 2.14. Statistical Analyses

The MTT assay data were analyzed using two-way analysis of variance (ANOVA) and the Student’s *t*-test was used for other experiments. GraphPad Prism version 5.03 (GraphPad Software, San Diego, CA, USA) was used to analyze and draw graphs. *p*-values less than 0.05 were considered statistically significant. All results were expressed as the mean ± standard deviation (SD).

## 3. Results

### 3.1. KLF2 Expression is Suppressed by EBV Infection in Gastric Carcinoma

**Figure 1:**
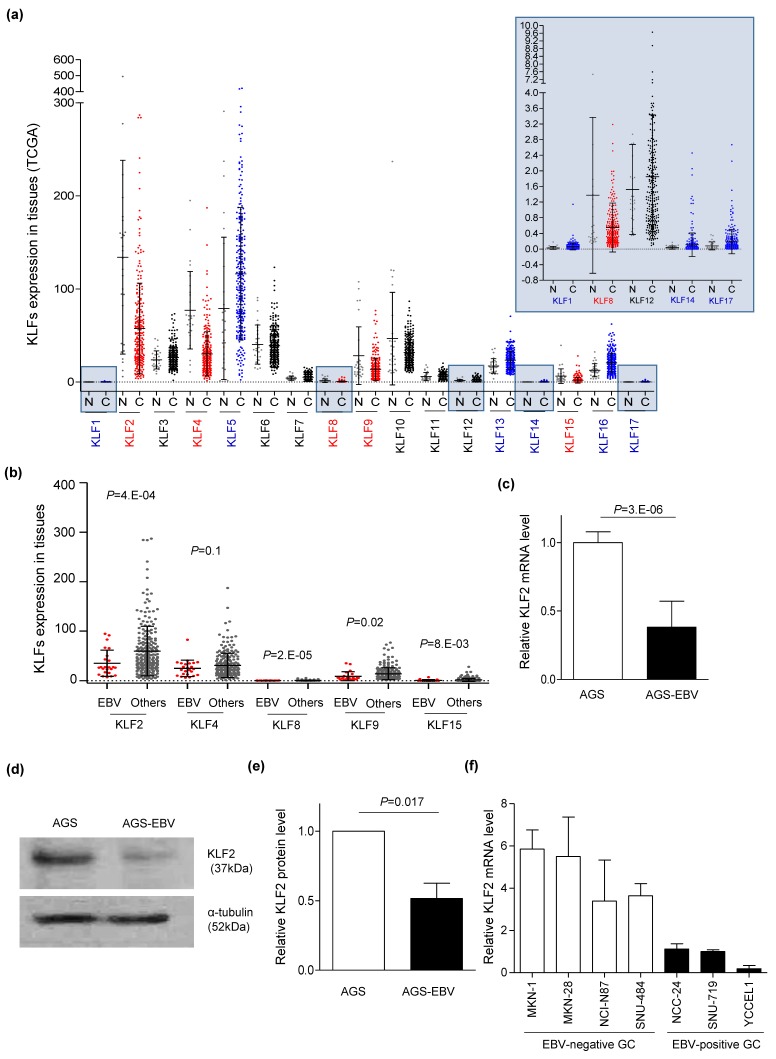
Suppressed Kruppel-like factor 2 (*KLF2*) expression by Epstein-Barr Virus (EBV) infection in gastric carcinoma. (**a**) RNA sequencing data from The Cancer Genome Atlas (TCGA) were used to analyze the expression of each KLF in normal tissues (N) and four subtypes of gastric cancer tissues (C). Significantly decreased (red) and increased (blue) KLF expression in C compared to N are indicated. Five KLFs with very low expression are shown at a larger scale at the upper right corner. (**b**) TCGA RNA sequencing data were used to select KLFs which showed reduced expression in gastric cancer tissues compared to normal tissues. KLF expression in EBV gastric cancer and in all the other three subtypes of gastric cancer (others) are shown for comparison. (**c**) mRNA level of *KLF2* was analyzed by qRT-PCR using a SYBR Green qPCR kit. (**d**) Western blot analysis was carried out to analyze the expression of *KLF2* using anti-KLF2 (1:500) antibody in three independent experiments. A representative result is shown. (**e**) Results of *KLF2* Western blot analysis for AGS-EBV cells conducted from three independent experiments were normalized using *α-tubulin* and expressed as ratios relative to the values obtained from AGS cells. Each value represents the mean ± standard deviation (SD) of three independent experiments. (**f**) *KLF2* mRNA levels in EBV-positive and EBV-negative cell lines were analyzed by qRT-PCR and shown as relative values to that of SNU-719. Each value represents the mean ± SD in three independent experiments.

To investigate the effect of EBV infection on Kruppel-like factor 2 (*KLF2*) expression in gastric cancer (GC) tissues, RNA sequencing data from TCGA were analyzed. The expression of five KLFs (*KLF2*, *4*, *8*, *9*, and *15*) was decreased more in GC tissues than in normal tissues (Figure 1a). We next examined whether the expression of these five KLFs was reduced in EBVaGC compared to other subtypes of GC (Figure 1b). In all four subtypes of GC, *KLF8* and *KLF15* had the least expression in GC tissues, while the expression of *KLF4* showed no significant differences between EBVaGC and the other GC subtypes. The expression of *KLF2* was the most reduced in EBVaGC compared to other subtypes of GC.

To identify whether the expression of *KLF2* was affected by EBV infection in GC cell lines, qRT-PCR was performed for *KLF2* using AGS and AGS-EBV cells, which differed only in EBV infection status. We found that the mRNA level of *KLF2* was 67% lower (Figure 1c) and the protein level of *KLF2* was 48.3% lower in AGS-EBV than in AGS (Figure 1d,e). In addition, qRT-PCR of *KLF2* was performed for EBV-negative (MNK1, MKN28, NCI-N87, and SNU-484) and EBV-positive (NCC24, SNU-719, and YCCEL1) cell lines. The results showed that the expression of *KLF2* was generally lower in the EBV-positive cell lines than in the EBV-negative cell lines (Figure 1f).

### 3.2. Screening EBV BART miRNAs that May Target KLF2

**Figure 2:**
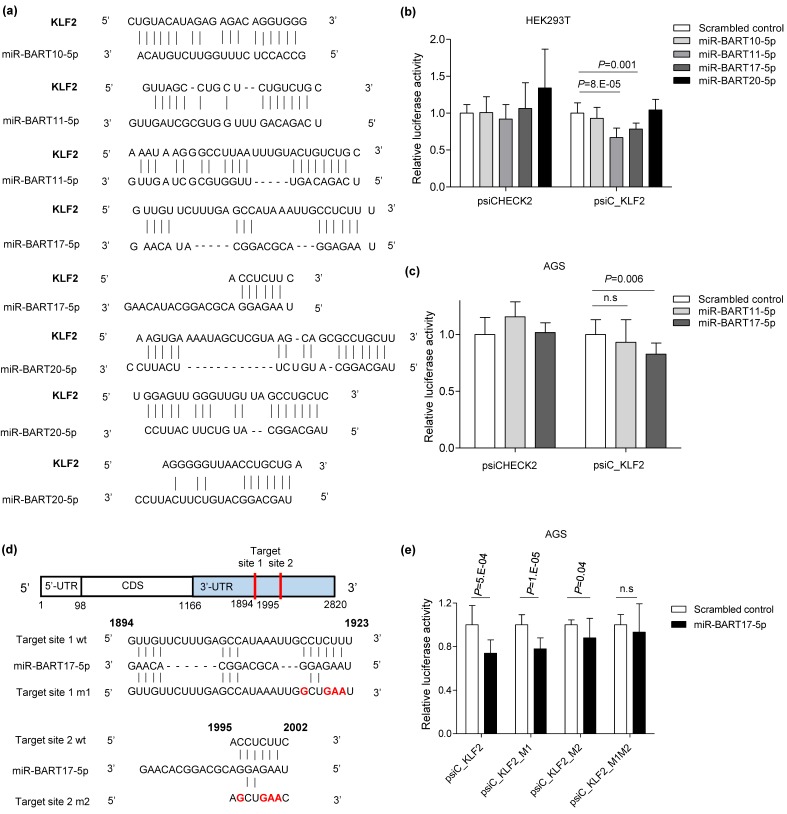
Screening of EBV microRNA that targets *KLF2*. (**a**) Seed matches between four BART miRNAs and the 3′UTR of *KLF2*. (**b,c**) psiC_KLF2 and each BART miRNA were co-transfected into HEK293T cells (**b**) and AGS cells (**c**). Luciferase reporter assays were measured 48 h after transfection. Three independent experiments were conducted to confirm reproducibility. Luciferase activity was normalized using internal firefly luciferase activity. The luciferase activity of the cells transfected with each BART miRNA is expressed as a ratio to the luciferase activity obtained from the scrambled control transfected cells. Error bars indicate SDs. (**d**) Schematic drawing shows the location of the predicted target sites 1 and 2 for miR-BART17-5p on the 3′UTR of *KLF2* mRNA (upper panel). Two sites in the 3′UTR of *KLF2* that seed match with miR-BART17-5p were mutated individually or together, and the mutated sequences are shown in red (lower panel). (**e**) Luciferase assay was carried out using AGS cells co-transfected with psiC_KLF2 (wildtype or mutants) and 30 nM miR-BART17-5p. Each value represents the mean ± SD in four independent experiments. Abbreviation: n.s, not significant.

We confirmed that *KLF2* expression was reduced in EBVaGC (Figure 1). In addition, previous studies have reported that BART miRNAs are significantly expressed in EBV latency type 1 [43]. To check if *KLF2* expression was suppressed by BART miRNAs in EBV-positive cells, an RNA hybrid program was used to predict BART miRNAs with the potential to target *KLF2*. Four BART miRNAs have seed match sequences for the 3’UTR of *KLF2*, and three BART miRNAs were expected to target more than two sites on the 3’UTR of *KLF2* (Figure 2a). To check whether these miRNAs directly target the *KLF2* 3’UTR, a luciferase reporter vector containing the 3′UTR of *KLF2* (psiC_KLF2) was constructed. psiC_KLF2 and each BART miRNA mimic were co-transfected and a luciferase assay was conducted. The results showed that luciferase activities were reduced when miR-BART11-5p (33%) or miR-BART17-5p (22%) was transfected (Figure 2b) into HEK293T cells. When AGS cells were co-transfected with psiC_KLF2 vector and either miR-BART11-5p or miR-BART17-5p mimics, luciferase activity was suppressed by miR-BART17-5p but not by miR-BART11-5p (Figure 2c). There were two miR-BART17-5p seed match sites in the 3′UTR of *KLF2* (Figure 2a). To determine whether they play important roles in reducing *KLF2* expression, one or both of the two seed match sites were mutated (Figure 2d). The psiC_KLF2 luciferase reporter vector with mutated site 1 (1894–1923) seed sequence was named psiC_KLF2_M1 and the vector with mutated site 2 (1995-2002) was named psiC_KLF2_M2. The vector containing both mutated 1 and 2 sites was named psiC_KLF2_M1M2. AGS cells were co-transfected with miR-BART17-5p and each of the vectors for a luciferase assay. While luciferase activity was reduced by 26.2% when the wild-type psiC_KLF2 was co-transfected with miR-BART17-5p; luciferase activity was less suppressed when psiC_KLF2_M1 (22.1%) or psiC_KLF2_M2 (12%) was co-transfected with miR-BART17-5p (Figure 2e). When psiC_KLF2_M1M2 was co-transfected, luciferase activity was not affected by miR-BART17-5p (Figure 2e). These results indicate that miR-BART17-5p binds to both of the sites and M2 is the major site to which miR-BART17-5p binds. 

### 3.3. miR-BART17-5p Suppresses Expression of KLF2.

**Figure 3:**
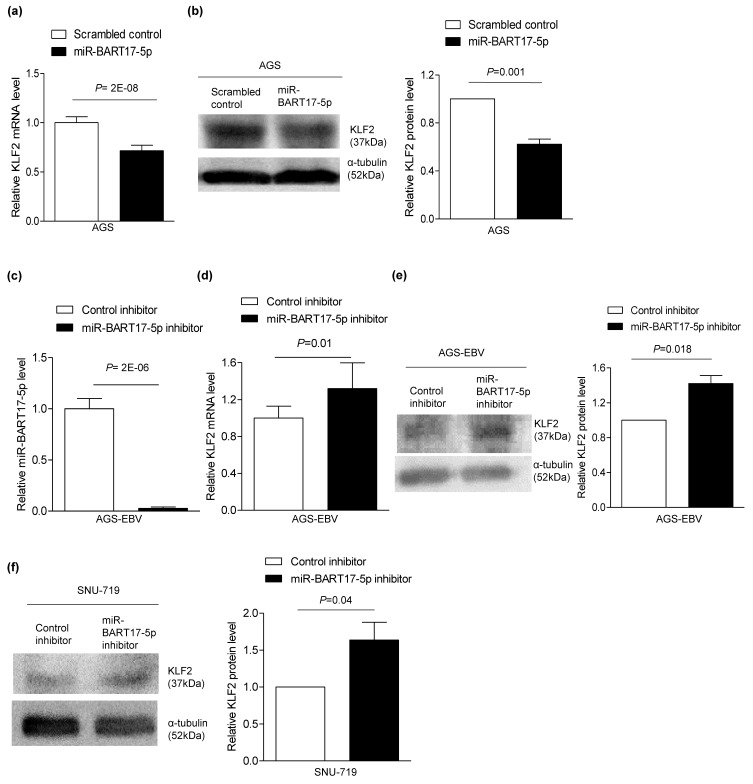
Expression of *KLF2* affected by transfection and inhibition of miR-BART17-5p. miR-BART17-5p mimic (30 nM) was transfected into AGS cells (**a,b**). (**a**) mRNA level of *KLF2* was analyzed by qRT-PCR using a SYBR Green qPCR kit. (**b**) Western blot analysis of *KLF2* expression using anti-KLF2 (1:500) antibody. Anti-α-tubulin antibody was used to confirm comparable loading (left panel). Western blot results have been normalized to *α-tubulin* and are expressed as ratios to the values obtained from the scrambled control. Error bars indicate SD (*n* = 3) (Right panel). AGS-EBV and SNU-719 were transfected with 30 nM miR-BART17-5p inhibitor (**c-f**). (**c**) miR-BART17-5p was analyzed by qRT-PCR in three independent experiments. Expression of miR-BART17-5p was normalized with U6 snRNA. (**d**) mRNA level of *KLF2* was analyzed by qRT-PCR using a SYBR Green qPCR kit. (**e**) Western blot analysis was performed in three independent experiments to measure *KLF2* protein level in AGS-EBV cells. Anti-α-tubulin antibody was used to confirm comparable loading (left panel). (**f**) Western blots were performed in three independent experiments to measure *KLF2* protein level in SNU-719 (left panel). Western blot results have been normalized to *α-tubulin* and are expressed as ratios to the values obtained from the control inhibitor. Error bars indicate SD (*n* = 3) (right panel).

To confirm that miR-BART17-5p suppresses *KLF2* expression, miR-BART17-5p was transfected into AGS cells. In AGS cells, the *KLF2* mRNA level was reduced by 29% (Figure 3a) and the protein level was reduced by 37% (Figure 3b), whereas the miR-BART17-5p inhibitor transfection in AGS-EBV cells not only suppressed the endogenous level of miR-BART17-5p (Figure 3c) but also increased mRNA (1.3-fold) (Figure 3d) and protein (1.4-fold) levels of KLF2 (Figure 3e). Furthermore, the miR-BART17-5p inhibitor increased the *KLF2* protein level by 1.6-fold in naturally EBV infected GC cell line, SNU-719 (Figure 3f). Therefore, miR-BART17-5p directly regulates *KLF2* expression and disrupts the expression of *KLF2* in EBVaGC cells.

### 3.4. miR-BART17-5p Promotes Migration and Anchorage-Independent Growth in AGS Cells

**Figure 4:**
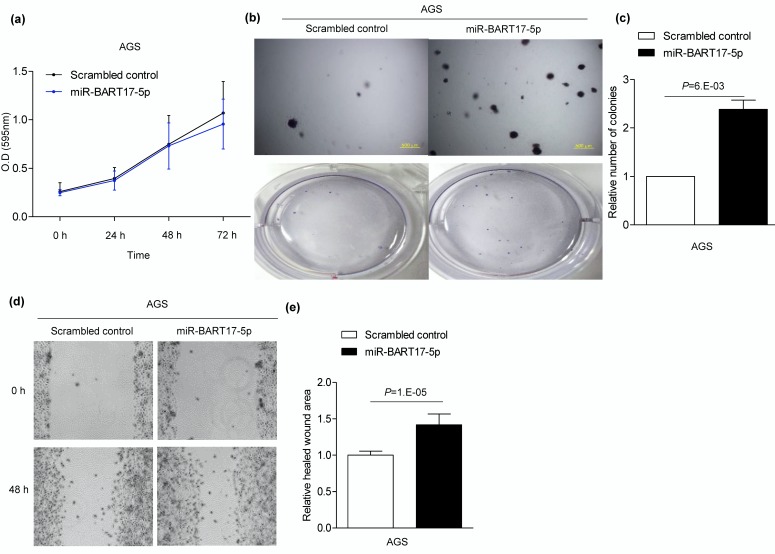
Enhanced AGS cell migration and anchorage-independent growth by miR-BART17-5p. AGS cells were transfected with 30 nM miR-BART17-5p mimic (**a–e**). (**a**) Before and every 24 h after miR-BART17-5p mimic transfection, 20 µl of MTT solution was added to each well to assess AGS cell proliferation. Experiments were conducted four times independently. (**b**) For conducting soft agar colony formation assay, transfected AGS cell was harvested after 24 h and seeded in agar. Cells placed in agar were observed after 4-6 weeks in three independent experiments. Upper panels were observed with microscope IX70 (Olympus, Tokyo, Japan) through a × 40 objective. Lower panels show the pictures taken with a camera. (**c**) Colonies were quantified using Image J software. Error bars indicate SD (*n* = 3). (**d**) Wound-healing assay was performed using AGS cells transfected with either miR-BART17-5p or the scrambled control. (**e**) Healed wound area was measured by Image J software. Error bars indicate SD (*n* = 3).

In previous results, miR-BART17-5p negatively regulated the expression of *KLF2*, therefore we tried to confirm the role of miR-BART17-5p in EBVaGC. To analyze the effect of miR-BART17-5p on cell proliferation, migration, and anchorage-independent growth, miR-BART17-5p was artificially delivered to EBV-negative AGS cells. Cell proliferation was not affected by miR-BART17-5p (Figure 4a). A soft agar colony formation assay showed that miR-BART17-5p promoted anchorage-independent growth by 2.1-fold compared to the scrambled control (Figure 4b,c). A wound-healing assay also showed that migration increased 1.3-fold when miR-BART17-5p was delivered to AGS cells compared with the scrambled control (Figure 4d,e).

### 3.5. miR-BART17-5p Inhibitor Suppresses Migration and Anchorage-Independent Growth in AGS-EBV Cells

**Figure 5:**
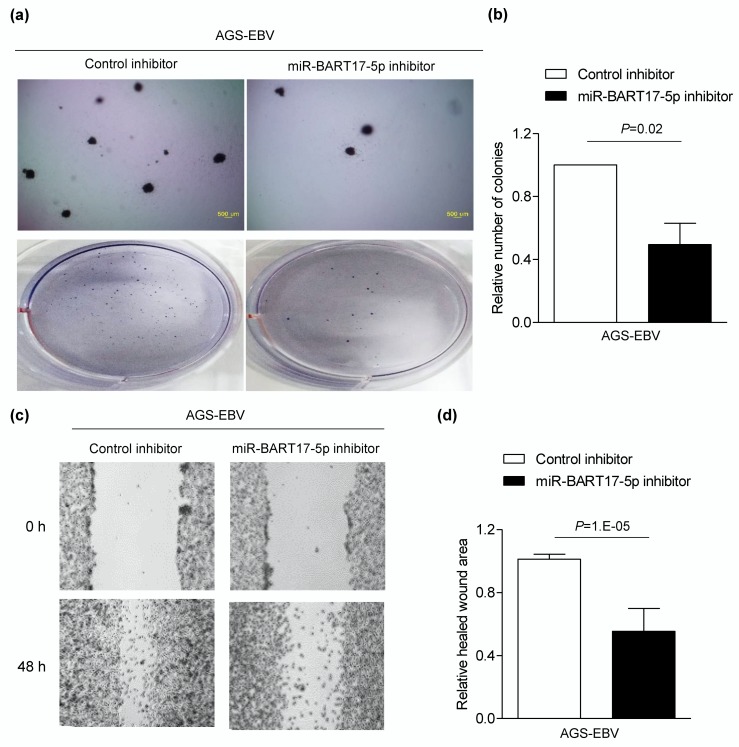
Suppressed AGS-EBV cell migration and anchorage-independent growth by the miR-BART17-5p inhibitor. AGS-EBV cells were transfected with the miR-BART17-5p inhibitor and all experiments were conducted three times independently (**a**–**d**). (**a**) To conduct soft agar colony formation assay, the transfected AGS-EBV cell was harvested after 24 h and seeded in agar. Cells placed in agar were observed after 4-6 weeks. All experiments were conducted three times. Upper panels were observed with microscope IX70 through a ×40 objective. Lower panels show the pictures taken with a camera. (**b**) Colonies were quantified using Image J software. Error bars indicate SD (*n* = 3). (**c**) Wound-healing assay was performed using AGS-EBV cells transfected with either the miR-BART17-5p inhibitor or the control inhibitor in three independent experiments. (**d**) Healed wound area was measured by Image J software. Error bars indicate SD (*n* = 3).

We then investigated the effect of endogenously expressed miR-BART17-5p on the tumorigenesis of AGS-EBV by delivering a miR-BART17-5p inhibitor into cells. A soft agar colony formation assay revealed that anchorage-independent growth was reduced by 50% following miR-BART17-5p inhibition (Figure 5a,b). In addition, wound-healing of AGS-EBV cells was hindered by 44.6% following transfection with the miR-BART17-5p inhibitor in comparison with the control inhibitor (Figure 5c,d). 

### 3.6. Knocking Down KLF2 Using siRNA Induces Migration and Anchorage-Independent Growth in AGS Cells

**Figure 6:**
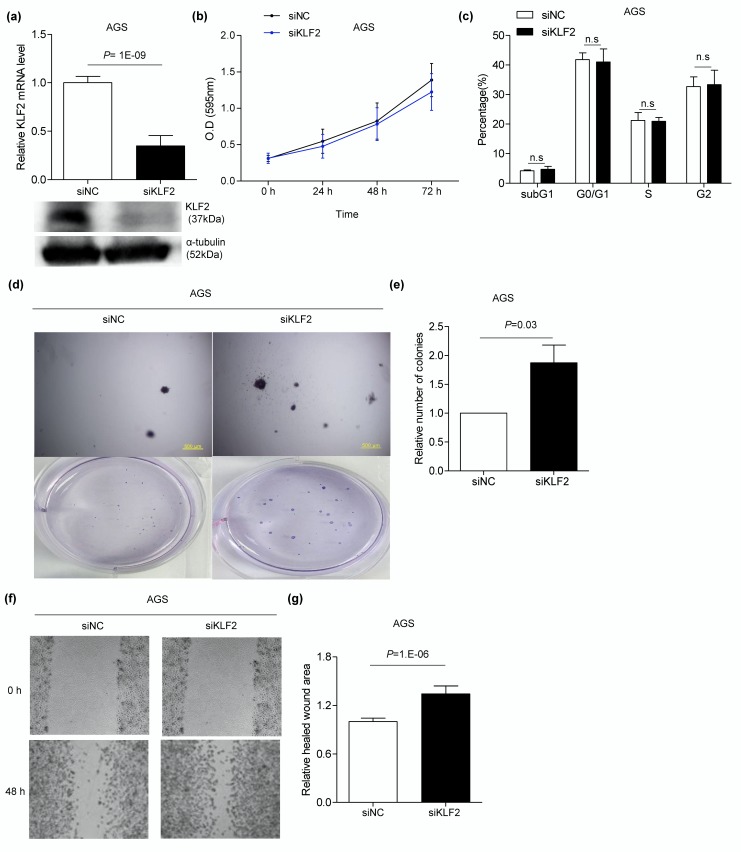
Promoted tumorigenesis of AGS cells through siKLF2. AGS cells were transfected with 30 nM siKLF2 (**a**–**g)**. (**a**) *KLF2* mRNA level was analyzed by qRT-PCR using a SYBR Green qPCR kit (Upper panel). Western blotting using anti-KLF2 (1:500) antibody showed expression of *KLF2*. Anti-α-tubulin antibody was used for loading control (lower panel). All experiments were conducted three times independently. (**b**) Before and every 24 h after siKLF2 transfection, 20 µl of MTT solution was added to each well to assess AGS cell proliferation in four independent experiments. (**c**) AGS cells were transfected with the negative control (siNC) or siKLF2. The proportion of the cells in each cell cycle phase was evaluated 48 h later by propidium iodide staining. All experiments were conducted four times independently. (**d**) To conduct soft agar colony formation assay, a transfected AGS cell was harvested after 24 h and seeded in agar. Cells placed in agar were observed after 4–6 weeks in three independent experiments. Upper panels were observed with microscope IX70 through a ×40 objective. Lower panels show the images taken with a camera. (**e**) Colonies were quantified using Image J software. Error bars indicate SD (*n* = 3). (**f**) Wound-healing assay was performed on AGS cells transfected with 30 nM siKLF2 to assess *KLF2* function in three independent experiments. (**g**) Healed wound area was measured by Image J software. Error bars indicate SD (*n* = 3). Abbreviation: n.s, not significant.

*KLF2* siRNA was transfected to confirm that the phenotype changes following miR-BART17-5p transfection were manifested by the downregulation of *KLF2*. *KLF2* expression in AGS was sharply decreased by siKLF2 transfection (Figure 6a) compared with control siRNA (siNC) transfection. After delivering the siKLF2, anchorage-independent growth of AGS on soft agar was increased 1.8-fold compared to cells transfected with the siNC (Figure 6d,e). Cell migration was also increased 1.3-fold by the siKLF2 compared with the siNC (Figure 6f,g). In contrast, cell proliferation and cell cycle were not affected following KLF2 knockdown using the siKLF2 (Figure 6b,c).

### 3.7. KLF2 Overexpression Inhibits Migration and Anchorage-Independent Growth in AGS-EBV Cells

**Figure 7:**
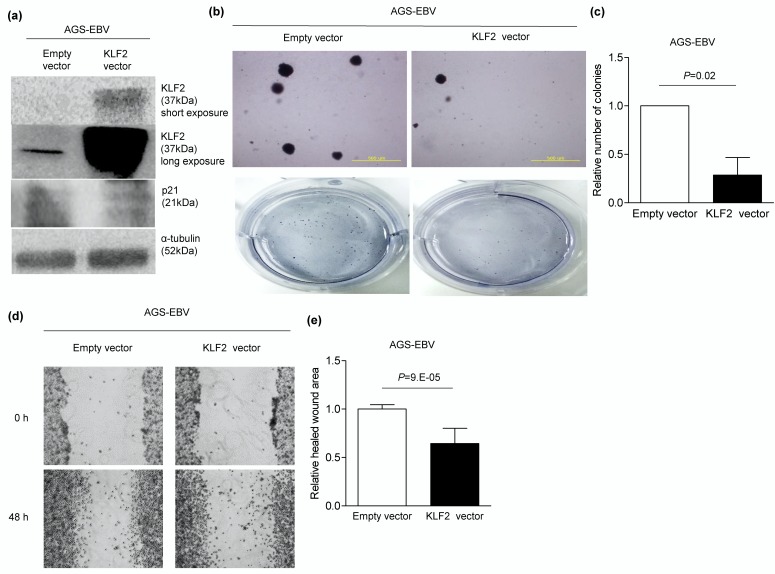
Inhibited tumorigenesis of AGS-EBV cells through *KLF2* overexpression. AGS-EBV cells were transfected with either the *KLF2* vector or the empty vector, and all experiments were conducted three times independently (**a**–**e**). (**a**) Western blot analysis was carried out using anti-KLF2 (1:500) antibody. Anti-p21 (1:500) detected the expression of *p21*, which is downstream of *KLF2*. Anti-α-tubulin antibody was used as a loading control. Long exposure was performed to show that *KLF2* was expressed even when the empty vector was delivered. (**b**) To conduct the soft agar colony formation assay, transfected AGS-EBV cells were harvested after 24 h and seeded in agar. Cells placed in agar were observed after 4-6 weeks in three independent experiments. Upper panels were observed with microscope IX70 through a ×40 objective. Lower panels show the images taken with a camera. (**c**) Colonies were quantified using the Image J software. Error bars indicate SD (*n* = 3). (**d**) Wound-healing assay was performed three times independently in AGS-EBV cells transfected with 500 ng of the *KLF2* expression vector or the empty vector. (**e**) Healed wound area was measured by Image J software. Error bars indicate SD (*n* = 3).

To confirm *KLF2* function, a *KLF2* overexpression vector was delivered to AGS-EBV, in which *KLF2* expression was lower than in AGS. Following *KLF2* overexpression (Figure 7a), anchorage-independent growth was reduced by 71% in AGS-EBV cells (Figure 7b,c). In addition, migration of AGS-EBV cells was suppressed by 36% compared to the control vector transfection (Figure 7d,e).

## 4. Discussion

We found that *KLF2* expression was lower in EBVaGC than in EBV-negative GC. In the process of testing the hypothesis that EBV miRNAs inhibit *KLF2* expression, we found that miR-BART17-5p directly targeted the *KLF2* 3′UTR to reduce mRNA and protein expression. We also found that miR-BART17-5p promoted cell migration and anchorage-independent growth by reducing the expression of *KLF2*.

The expression of miR-BART17-5p increases in plasma with the progression of NPC tumors [44]. miR-BART17-5p is also a biomarker that is associated with poor prognosis, since it is only detected in the serum of patients with recurrent NPCs [17]. Although there is a considerable expression of miR-BART17-5p in EBVaGC [20,21], little is known about the role of miR-BART17-5p in EBVaGC. In NPC, *LMP1* has been reported to be a target of miR-BART17-5p [9]. As *LMP1* is rarely detected in EBVaGC [45,46,47], miR-BART17-5p may target *LMP1* and almost abrogate *LMP1* expression in EBVaGC. Even when miR-BART17-5p is bound to the 3′UTR of cellular N-myc downstream-regulated gene 1 (*NDRG1*) [11], miR-BART17-5p did not affect *NDRG1* expression, curiously [48]. Our study shows that highly expressed miR-BART17-5p targets *KLF2* in EBVaGC, revealing the role of this BART miRNA.

Although *KLF2* acts as a tumor suppressor in several cancers, including GC [49,50], reports show that *KLF2* acts as an oncogene in liver cancer [51]. Our results support that *KLF2* is a target of miR-BART17-5p and acts as a tumor suppressor in EBVaGC. Even though *KLF2* expression in GC tissues including EBVaGC were not tested in this study, we analyzed EBVaGC patient data from TCGA [3]. We found that *KLF2* expression was lower in GC than in normal tissues, and that *KLF2* expression was lower in EBVaGC than in other GCs (Figure 1a,b). In addition, all three naturally EBV infected GC cell lines as well as AGS-EBV cells showed low-level *KLF2* expression (Figure 1f). This is consistent with microarray data showing a greater reduction in the expression of *KLF2* in EBV-infected cell lines than in EBV-uninfected cell lines [10]. Thus, *KLF2* may be expressed at low levels in EBVaGC.

In previous studies, EBV infection in gastric epithelial and gastric carcinoma cell lines increased anchorage-independent growth despite the restricted viral protein expression [10,52]. It was speculated that this phenotype is due to BART miRNAs. Wang et al. [53]. showed that miR-BART7 promoted anchorage-independent growth. Additionally, several studies showed that BART miRNAs promoted cell migration [54,55]. Based on our *KLF2* knockdown and overexpression data, the phenotype induced by miR-BART17-5p may be due to the decreased expression of *KLF2*. Previous reports showed that *KLF2* suppressed anchorage-independent growth by inhibiting the expression of *Gli1* in liver cancer [56] as well as migration by inhibiting *MMP2*, *N-cadherin,* and *vimentin* in several cancers [48,49]. In our study, reducing *KLF2* expression may have caused anchorage-independent growth and cell motility through the mechanisms described above. 

In the present study, the expression level of *KLF2* in AGS-EBV was lower than the level of *KLF2* following miR-BART17-5p transfection to AGS. This suggests that not only miR-BART17-5p but also other EBV genes may down-regulate *KLF2* expression. The expression of *EZH2*, a histone methyltransferase, is increased in NPC [57]. EBV infection in primary B cells induced *EZH2* expression [58]. In addition, RNA sequencing data from TCGA showed that the expression of *EZH2* in EBVaGC was higher than in normal tissues and other subtypes of GC [3]. Since *KLF2* is silenced by *EZH2* in GC [27], increased expression of *EZH2* in EBVaGC may have silenced *KLF2*. Li et al. [59] reported that Helicobacter pylori (*H. pylori*) induced miR-25 to reduce the expression of *KLF2*. As GC can be co-infected with EBV and *H. pylori*, both miR-25 and miR-BART17-5p may exert a simultaneous effect on the expression of *KLF2*. It is not clear which pathway would be more important for the development of GC based on our data, as we did not test the effect of *H. pylori* infection. Further studies would be required to clarify this point.

Although in a previous study *KLF2* was reported to be a tumor suppressor that affected cell proliferation in GC [34], cell proliferation was not affected by either siKLF2 or miR-BART17-5p in the present study. *KLF2* was shown to inhibit cell proliferation by increasing the expression of *p21*, a cell cycle blocker [60]. However, siKLF2 transfection did not affect the cell cycle and the *KLF2* overexpression vector transfection did not affect the expression of *p21* in our experiments. Recently, a study reported that *FOXO4* binds to the activation domain of *KLF2* and co-operates to induce *p21* expression [61]. RNA sequencing data from TCGA showed that *FOXO4* is decreased in EBVaGC [3]. This is consistent with a previous study where the expression of *FOXO4* was reduced by *LMP1* and *LMP2A* in AGS-EBV [62]. Therefore, even when *KLF2* was overexpressed in AGS-EBV cells, *p21* may not be induced due to low *FOXO4* expression in the cells. In addition, we previously reported that miR-BART17-5p did not affect cell proliferation, which is consistent with this study [63]. Many studies suggest that the growth rate of cancer cells does not necessarily correlate with cell migration and invasion abilities [64,65]. Thus, we propose that miR-BART17-5p may play a role in the metastatic process rather than in tumor growth of EBVaGC.

## 5. Conclusions

Our data suggest that miR-BART17-5p, which is highly expressed in EBVaGC, plays an oncogenic role by inhibiting a tumor suppressor *KLF2* expression. miR-BART17-5p increased cell motility and anchorage-independent growth, features associated tumor metastasis. Therefore, miR-BART17-5p may serve as a potential therapeutic target of EBVaGC. Inhibitors of miR-BART17-5p may be useful alone or in combination with other therapeutic agents to treat EBVaGC.
